# Highly Stable PEGylated Poly(lactic-co-glycolic acid) (PLGA) Nanoparticles for the Effective Delivery of Docetaxel in Prostate Cancers

**DOI:** 10.1186/s11671-016-1509-3

**Published:** 2016-06-21

**Authors:** Long-Bin Cao, Sha Zeng, Wei Zhao

**Affiliations:** Department of Urology, Liaocheng Hospital, 67, Dongchang Xi Road, Liaocheng, Shandong 252000 People’s Republic of China; Central Laboratory, Liaocheng Hospital, Liaocheng, Shandong 252000 People’s Republic of China

**Keywords:** LHRH, Polymeric micelles, Docetaxel, Prostate cancers, Anticancer effect, Targeted delivery

## Abstract

In the present study, a highly stable luteinizing-hormone-releasing hormone (LHRH)-conjugated PEGylated poly(lactic-co-glycolic acid) (PLGA) nanoparticles were developed for the successful treatment of prostate cancers. We have demonstrated that a unique combination of targeted drug delivery and controlled drug release is effective against prostate cancer therapy. The docetaxel (DTX)/PLGA-LHRH micelles possessed a uniform spherical shape with an average diameter of ~170 nm. The micelles exhibited a controlled drug release for up to 96 h which can minimize the non-specific systemic spread of toxic drugs during circulation while maximizing the efficiency of tumor-targeted drug delivery. The LHRH-conjugated micelles showed enhanced cellular uptake and exhibited significantly higher cytotoxicity against LNCaP cancer cells. We have showed that PLGA-LHRH induced greater caspase-3 activity indicating its superior apoptosis potential. Consistently, LHRH-conjugated micelles induced threefold and twofold higher G2/M phase arrest than compared to free DTX or PLGA NP-treated groups. Overall, results indicate that use of LHRH-conjugated nanocarriers may potentially be an effective nanocarrier to effectively treat prostate cancer.

## Background

Prostate cancer (PCa) is one of the most frequently diagnosed cancers in men (especially Western men) [1]. As per the figures available from American Cancer Society, nearly 230,000 new cases have been identified in the USA alone in year 2014. This statistics is rapidly increasing with every passing year. PCa has surpassed heart disease as the top killer of men over the age of 85 years [2, 3]. Although the mortality rate due to prostate gland cancer is decreasing, the cost of therapy and surgery makes it costly for a common man [4]. At present, chemotherapy and radiation therapy are the treatments of choice for PCa; however, current therapies are highly toxic to normal cells/tissues. Especially, conventional chemotherapy results in off targeting that could cause severe damage to rapidly dividing cells, and patient often experiences organ toxicity [5, 6].

In this regard, docetaxel (DTX), a taxane, remains an important class of antitumor agents, effective against advanced prostate cancer therapy, and until recently, only DTX-based chemotherapy is shown to be efficacious [7, 8]. However serious side effects such as myelosuppression, neurotoxicity, acute hypersensitivity reactions, nasolacrimal duct stenosis, and febrile neutropenia limit its clinical applications [9]. Additionally, solubility-related side effects further hinder its pharmacological action, and impaired drug delivery to tumor cells have also been shown to promote resistance to DTX. Therefore, an effective delivery system has to be developed to mitigate the toxicity of DTX and to improve its chemotherapeutic efficacy against cancers [10].

Nanoparticles loaded with various therapeutic agents act as drug carriers that can circulate in the blood longer, increase drug concentrations in tumors, and thus improve the chemotherapeutic efficacy in respective cancers. In this regard, the number of drug delivery strategies has been developed till date to encapsulate drug in carriers such as polymer nanoparticles (NPs), liposomes, inclusion complex, and self-assemblies utilizing their complex molecular structures [11, 12]. Of all, poly(lactic-co-glycolic acid) (PLGA)-based nanoparticulate systems are reported to be non-toxic, biodegradable, and non-immunogenic and thus add great valuable to medical applications. Many PLGA-based formulations are in various phases of clinical trial [13]. The PEGylation of PLGA NP prevents the opsonin binding, prolongs the circulation time of nanosystems, and could reduce the rapid uptake of reticuloendothelial system (RES) in the blood. Again, such construct could take advantage of enhanced permeability and retention (EPR) effect and accumulate preferentially in tumors [14]. Targeted nanotherapies may improve therapeutic outcome of prostate cancer treatment by selectively targeting the receptor overexpressed in the cancer cells. Active tumor-targeting ability could be obtained by conjugating certain types of ligands which are specific towards the receptor present in the cancer cells [15]. This will pave way for high accumulation and sustained release of therapeutic molecule in the tumor tissues. In PCa, many receptors are overexpressed including luteinizing-hormone-releasing hormone (LHRH), prostate-specific membrane antigen (PSMA), and epidermal growth factor receptor (EGFR) receptors [16]. Of all LHRH, a 10-amino-acid peptide hormone is the most interesting. LHRH is detected in ~85 % of PCa and has low expression in normal cell types. Recently, LHRH and its synthetic analogs are frequently used in the management of PCa. Especially, LHRH-R which is the synthetic analog (to circumvent the short half-life of natural LHRH), has been used in the present study [17].

In this study, LHRH-conjugated PEGylated PLGA nanoparticle system was developed to encapsulate and deliver the DTX to the prostate cancer site. We hypothesized that LHRH-conjugated nanoplatform would increase the therapeutic efficacy of DTX towards prostate cancers. For this purpose, PEG-PLGA polymer block was synthesized which was then conjugated with LHRH using chemical reactions. Entrapment, release, and cytotoxicity of DTX were assessed to understand the effect of controlling drug release patterns on cellular response to DTX-loaded LHRH-based polymeric nanoparticles. Cell apoptosis assay and cell cycle analysis were performed to further prove the targeting ability of ligand-directed nanoparticles towards prostate cancers.

## Methods

### Materials

PLGA (lactide/glycolide ratio of 50:50, carboxylic acid end group, molecular weight, 17,000 Da) was purchased from Sigma-Aldrich, China. The heterofunctional PEG polymer with terminal amine and carboxylic acid functional group (NH2-PEG-COOH) was procured from JenKem Technology USA. LHRH analog peptide (1185 g/mol) (PYR-His-Trp-Ser-Tyr-DLys-Leu-Arg-Pro-Gly) was purchased from Hanhong Group (Shanghai, China). Docetaxel was obtained from Sigma-Aldrich, China. All other chemicals were reagent grade and used as such.

### Formulations of Docetaxel-Loaded LHRH-Conjugated PEGylated PLGA Nanoparticle

#### PLGA-PEG-COOH Conjugations

Approximately, 3 g of PLGA-COOH was dissolved in anhydrous methylene chloride, and to this organic solution, 70 mg of *N*-hydroxysuccinimide (NHS) and 140 mg of 1-ethyl-3-(3-dimethylaminopropyl)-carbodiimide (EDC) was added. The organic mixture was stirred continuously for 12 h at room temperature. PLGA-NHS was obtained by precipitation with cold diethyl ether (10 mL) as a white solid, which was filtered and repeatedly washed in a cold mixture of diethyl ether and methanol and then dried with nitrogen and under vacuum (yield, ∼78 %). The activated PLGA-NHS (3 g) was dissolved in anhydrous chloroform, and to this organic solution, 0.75 g of NH2-PEG-COOH and 40 mg of *N*,*N*-diisopropylethylamine was added and stirred for 24 h. The resulting diblock polymer was precipitated by the addition of ice-cold diethyl ether, washed, and dried.

#### PLGA-PEG-LHRH Conjugations

LHRH-conjugated diblock polymer was synthesized by the addition of 100 mg of PLGA-PEG-COOH and 25 mg of LHRH-NH2 to anhydrous methylene chloride solution. The organic mixture was stirred for 45 min, following which 3 mg of EDC and 1 mg of 4-dimethylaminopyridine were added. The mixture was stirred for 48 h, purified with dialysis, and then lyophilized.

#### Preparation of Drug-Loaded Nanoparticles

DTX-loaded PLGA nanoparticles were prepared by modified solvent-evaporation method [18]. Briefly, 15 mg of DTX and 50 mg of PLGA-PEG-LHRH copolymer was dissolved in 6 ml of chloroform and stirred for 30 min. The organic mixture was emulsified by adding to water (50 ml) in a drop-wise manner. The resulting o/w emulsion was allowed to be stirred (1000 rpm) for 3 h, and the resulting core-shell nanoparticle was separated by dialysis method (against water) to isolate it from the residual surfactants and unencapsulated drug and were washed with distilled water. The excess-free drug was separated by ultracentrifugation (CS150NX, Hitachi, Japan) at high speed (500*g*-force) for 20 min. The supernatant was used to calculate the amount of drug entrapped in the PLGA NP.

### Particle Size Analysis

The particle size and size distribution were determined by dynamic light scattering technique using Zetasizer NanoZS, Malvern Instruments Ltd., Malvern, UK. The samples were diluted suitably such that the mean count rate was approximately around 250. Each sample was measured in triplicate.

### Drug Loading and Encapsulation Efficiency

The amount of drug entrapped in the nanoparticle was calculated by dissolving the freeze-dried NP in methanol and sonicated for 20 min [19]. The solution was filtered, and the filtrate was used to analyze the amount of drug entrapped.

$$ \mathrm{D}\mathrm{L}\ \left(\%\right) = \left(\mathrm{Weight}\ \mathrm{of}\ \mathrm{drug}\ \mathrm{in}\ \mathrm{N}\mathrm{P}/\mathrm{Weight}\ \mathrm{of}\ \mathrm{N}\mathrm{P}\right) \times 100 $$

$$ \mathrm{E}\mathrm{E}\ \left(\%\right) = \left(\mathrm{Actual}\ \mathrm{weight}\ \mathrm{of}\ \mathrm{drug}/\mathrm{Theoretical}\ \mathrm{weight}\ \mathrm{of}\ \mathrm{drug}\right) \times 100 $$

### Morphology

The morphological examination of DTX/PLGA-LHRHwas carried out through a high-resolution transmission electron microscopy (TEM, Hitachi® 800MT, Japan). The liquid samples were counterstained with phosphotungistic acid and placed over a carbon-coated copper grid and air-dried.

### Release Study

The release of DTX in phosphate-buffered saline was evaluated by a dialysis method. For this purpose, 1 mL of DTX/PLGA-LHRH and DTX/PLGA NP suspension (2 mg equivalent of DTX) were transferred into a dialysis tube (molecular weight cutoff 3000, Membra-Cel®, Viskase, USA), sealed, and placed in a tube containing 20 ml of release medium. The whole assembly was kept in a horizontal laboratory shaker (100 rpm) and maintained at 37 °C. At specific time intervals, 1 ml of sample was removed and replaced with equal amount of fresh release medium. The amount of drug present in the release media was analyzed using a HPLC method. A Shimadzu 510 HPLC instrument consisted of BDS reverse phase column (150 × 4.6 mm × 5), Shimadzu 486 tunable absorbance UV detector, and SPD-10A detector, and a 20-μL Rheodyne injection syringe was used. A freshly prepared mixture of methanol and water (70:30 *v*/*v*) was used as a mobile phase. Cumulative amount of drug released was evaluated as the percentage of total drug release to the initial amount.

### In Vitro Cytotoxicity Assay

Human prostate cancer cell and lymph node prostate adenocarcinoma (LNCaP) was purchased from American Type Culture Collection (ATCC, Manassas, VA). LNCaP was grown in RPMI 1640 culture media supplemented with 10 % FBS and 1 % penicillin/streptomycin mixture. The cells were incubated in ambient conditions of 37 °C and 5 % CO2.

For cytotoxicity assay, 1 × 10^4^ cells were seeded in a 96-well plate and allowed to attach for overnight. The following day, the media was replaced with fresh media and cells were exposed with blank polymers, free DTX, DTX/PLGA NP, and DTX/PLGA-LHRH NP and incubated for 24 h. At the end of the experiment, the media was removed and washed twice with PBS. The fresh media containing 0.5 mg/mL MTT (3-(4,5-dimethylthiazol-2-yl)-2,5-diphenyltetrazolium bromide) was added and incubated for additional 3 h. The formazan crystal was extracted with the addition of 100 μl of DMSO. The wavelength was studied at 570 nm using a microplate reader (iMark, Bio-Rad, USA).

### Caspase-3 Activity

Caspase-3 activity was analyzed with Caspase-Glo 3 assay kit as per manufacturer’s protocol. Cells were processed as mentioned above, and the cells were then extracted by the addition of 0.25 % Trypsin-EDTA digestion. The cells were centrifuged and washed twice and re-suspended in PBS buffer. Caspase-Glo was added to the cells and incubated for 1 h at room temperature. The cell suspension was then transferred to culture tubes and determined by luminometer.

### Cellular Uptake Study

Flow cytometry was utilized to examine the cellular uptake of the targeted and non-targeted NPs. Rhodamine-B was loaded in PLGA NP to observe the cellular uptake in LNCaP prostate cancer cells. For this, the cells were seeded in 6-well plate, and after overnight incubation, the cells were exposed with PLGA and PLGA-LHRH formulation and incubated for different time points. The cells were then washed and extracted by trypsinization process. The cells were then washed again with PBS (two times), and the cells were then suspended in a flow cytometry buffer. The amount of NP internalization was determined using flow cytometer (BD FACSCalibur equipped with 488 and 633 nm lasers) in the FL2 channel. The experiments were run in triplicate and repeated three times.

### Cell Cycle Analysis

The cells were seeded in 6-well plate and treated with respective formulations and further incubated for 24 h. The cells were then collected by trypsinization and centrifuged at 1500 rpm for 2 min. The cells were then washed with ice-cold PBS buffer and stained with propidium iodide and ribonuclease for 30 min. The cell cycle was analyzed by flow cytometer (FACSCalibur flow cytometer; BD Biosciences).

### Statistical Analysis

Data were expressed as mean ± standard deviation from triplicate experiments and analyzed with IBM SPSS (v 13.0; SPSS Inc, Chicago, IL). *P* < 0.05 was considered statistically significant.

## Results and Discussion

**Fig. 1:**
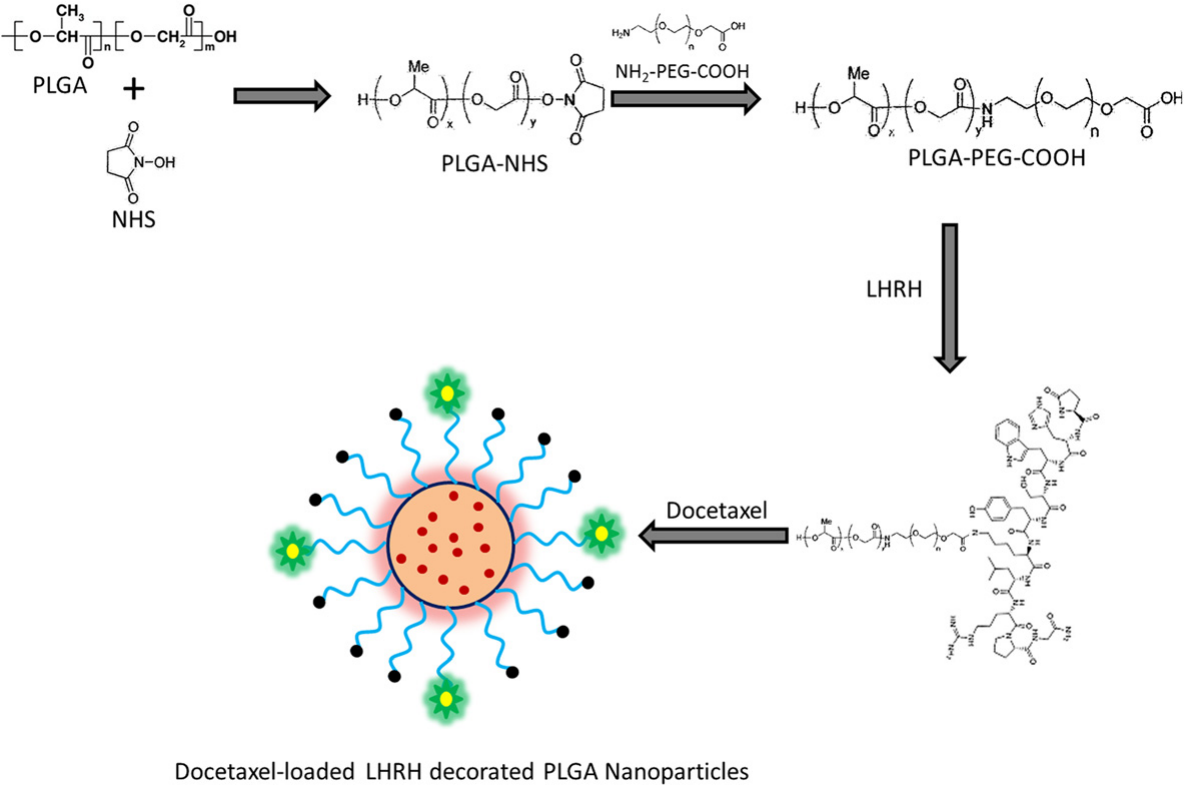
Schematic illustration of formulation of docetaxel-loaded polymeric micelles. PLGA-PEG was conjugated with LHRH moiety via standard carbodiimide/NHS-mediated chemistry. The PLGA-PEG-LHRH and docetaxel was self-assembled to form core-shell nanoparticles

Chemotherapy and radiation therapy are the treatment of choice for PCa. However, these therapies are highly toxic to normal cells/tissues limiting its further clinical application. Docetaxel (DTX), a taxane, is indicated in advanced prostate cancer therapy as a first-line therapy agent, and therefore, an effective delivery system has to be developed to mitigate the toxicity of DTX and to improve its chemotherapeutic efficacy against cancers. The present study is an attempt to encapsulate and deliver the DTX to the prostate cancer site using a novel LHRH-conjugated PEGylated PLGA nanoparticle system. We hypothesized that LHRH-conjugated nanoplatform would increase the therapeutic efficacy of DTX towards prostate cancers. LHRH, which is a 10-amino-acid peptide hormone is detected in ~85 % of PCa and have low expression in normal cell types. For this purpose, PLGA-COOH was activated by carbodiimide/NHS-mediated chemistry and then conjugated with heterofunctional PEG, NH2-PEG-COOH. The diblock polymer PLGA-PEG-COOH was then conjugated with LHRH (Fig. 1).

### Particle Size and Morphological Analysis

**Fig. 2:**
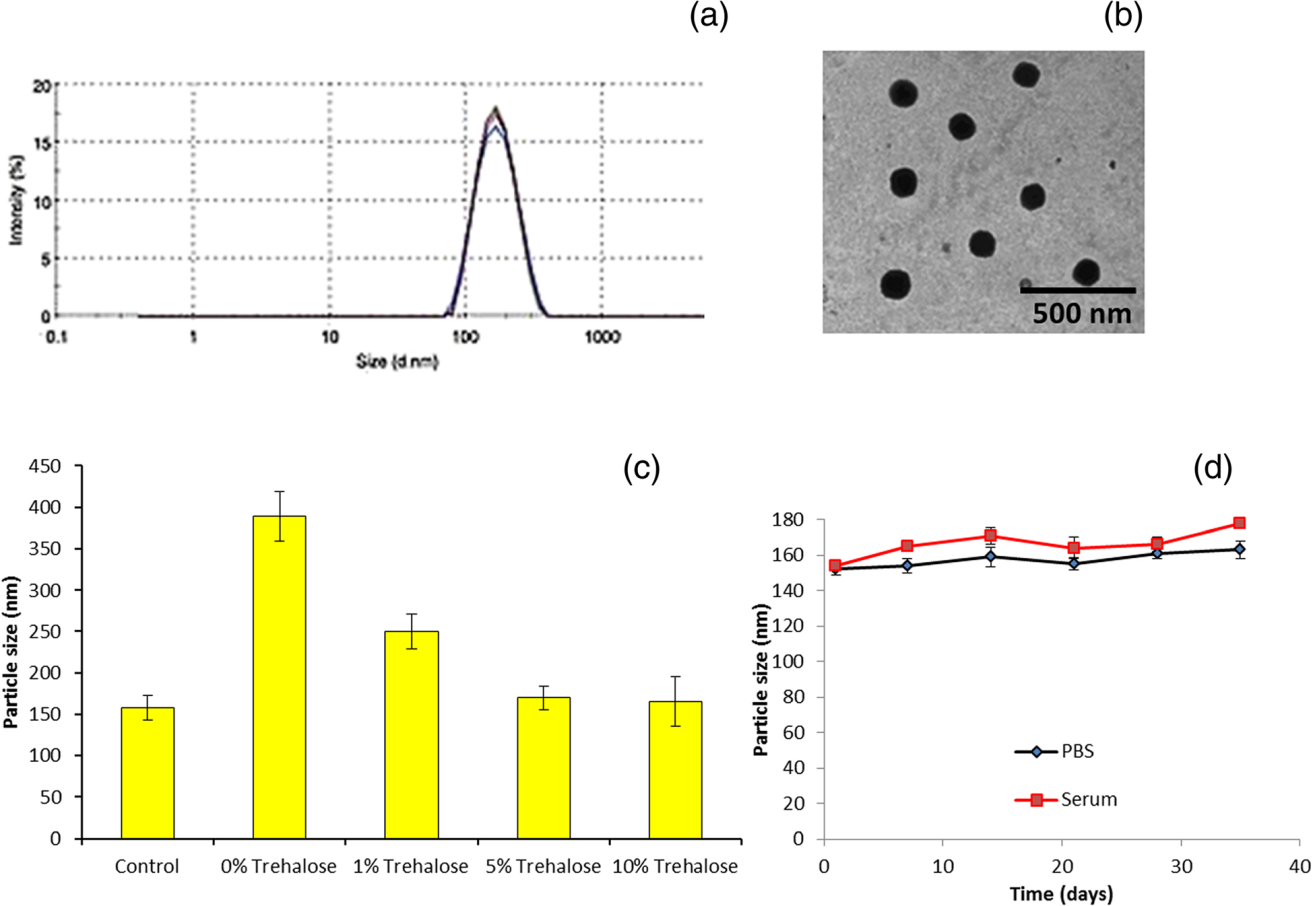
**a** Particle size distribution of PLGA-LHRH micelles measured by dynamic light scattering technique. **b** Transmission electron microscope image of PLGA-LHRH. **c** Solid state stability analysis. **d** Storage stability study

The DTX and PLGA-PEG-LHRH were self-assembled to form a stable drug-loaded micellar architecture. The particle size of PLGA-LHRH was found to be around ~170 nm with an excellent dispersity index of 0.15 (PDI) (Fig. 2a). A size of less than <200 nm is clinically relevant for passive tumor targeting via EPR [20]. To study the stability of the PLGA-LHRH micelles, the size and size distribution of the freshly prepared micelles and micelles stored in 4 °C for 1–2 months were also observed (data not shown). The size was almost the same even when stored for a prolonged period of time. Here in this study, surface modifications of PLGA NP with PEG are expected to reduce non-specific interactions with RES systems and prolong the blood circulation time.

The TEM image clearly showed spherical nature of the drug-loaded micellar structures. The average diameter ranged from 150 to 200 nm and uniformly dispersed on the carbon-coated copper grids, and no aggregation was observed (Fig. 2b). The sizes of the drug-loaded micelles measured by TEM were smaller than those measured by DLS because TEM measures the size of the dried micelles while DLS measures the hydrodynamic diameters of the micelles. The stability was further assessed by freeze drying process. The nanoparticles were freeze dried with various amounts of trehalose (as a cryoprotectant) and then reconstituted (Fig. 2c). The particle size significantly increased in the absence of cryoprotectant while addition of trehalose did reduce the size below 300 nm; 5 % trehalose was observed to be optimal as the size of particles were almost equal to that of control (liquid dispersion) while further increase in the percentage of trehalose did not have any influence. The nanoparticles also exhibited a long-term storage stability indicating its excellent stability (Fig. 2d).

The amount of drug entrapped in the micelles was determined by HPLC technique. The entrapment efficiency was more than 85 % while the actual drug loading was more than >25 % *w*/*w*. The high drug loading capacity of delivery system has clinical relevance.

### In Vitro Drug Release Study

**Fig. 3:**
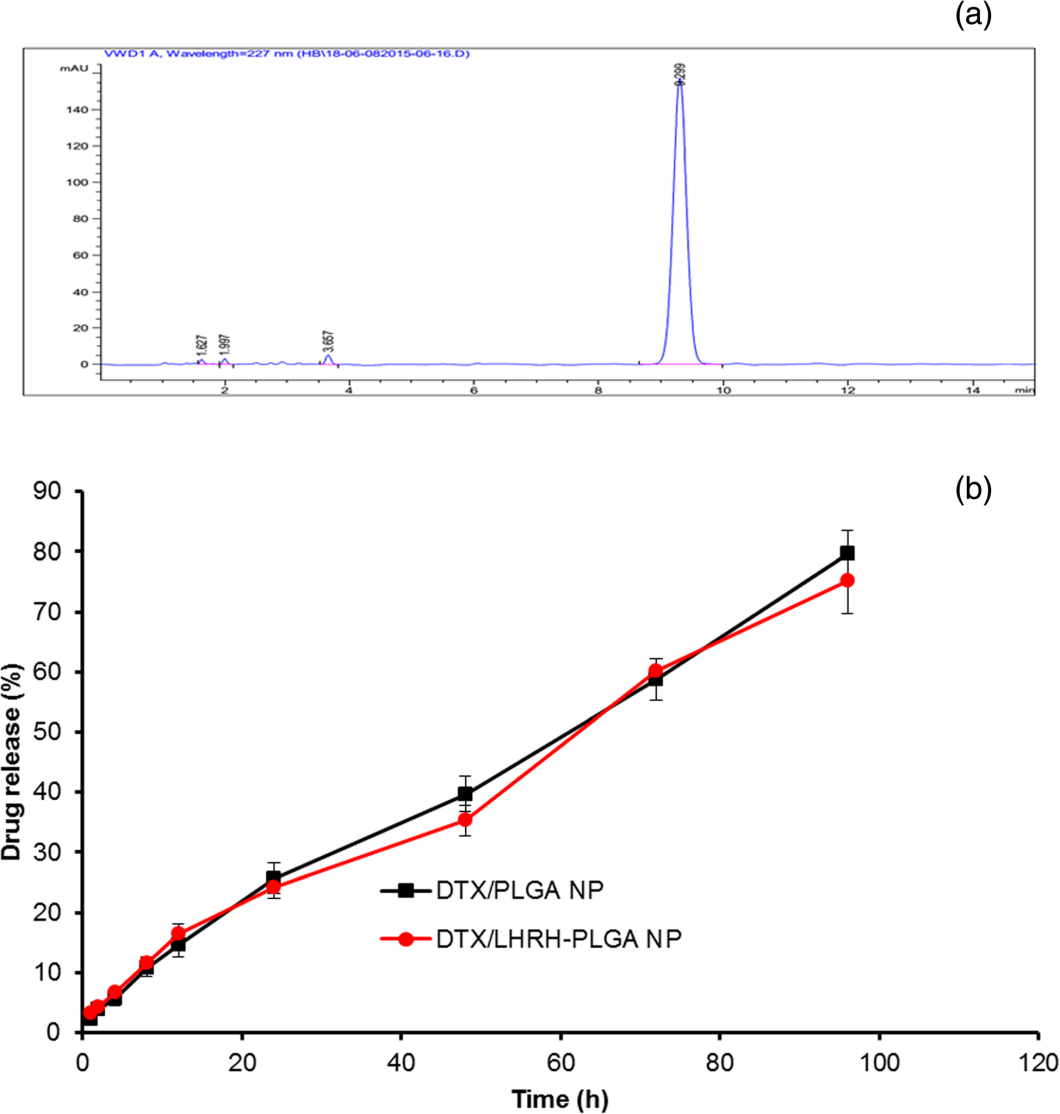
**a** HPLC chromatogram of DTX. **b** In vitro release profile of DTX from PLGA and PLGA-LHRH micelles. The release study was performed in phosphate buffered saline (PBS; pH 7.4) at 37 °C

The release profile of DTX (Fig. 3a) from PLGA and PLGA-LHRH micelles were studied at phosphate-buffered saline (pH 7.4) at 37 °C. As shown in Fig. 3b, a sustained release of DTX was observed from the micelles throughout the study period. No sign of initial burst release was observed indicating that the drug was stably incorporated in the core of the micelles. Similarly, LHRH conjugation also has no effect on the overall release rate as the release from both the micelles was almost similar throughout the study period. For example, nearly ~25 % of DTX released from both the micelles at the end of 24 h, while nearly 80–85 % of drug release by the end of 96 h of study period. Such release profile will be very efficacious from tumor targeting perspective. A delivery system which can release the drug in a controlled rate in the physiological conditions is most desirable for the tumor targeting as it will have greater chance to accumulate in the tumor tissues and effectively minimize the drug-related systemic toxicity [21].

### Cellular Uptake

**Fig. 4:**
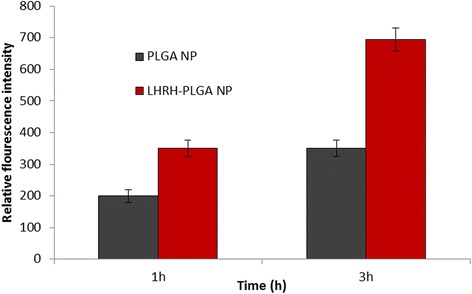
Cellular uptake of PLGA NP and PLGA-LHRH micelles in LNCaP prostate cancer cells. Cellular uptake was recorded byflow cytometry analysis on a BD FACS caliber. Rhodamine-B was used as a fluorescent moiety and incorporated in the nanocarriers

The effect of LHRH was investigated by studying the cellular internalization of rhodamine-B loaded targeted and non-targeted PLGA micelles in LNCaP cancer cells. LNcaP is reportedly overexpress LHRH receptors than when compared to normal cell lines [22]. In this study, the cellular uptake of rhodamine-loaded non-conjugated or conjugated micelles was evaluated by flow cytometer analysis. As shown in Fig. 4, significant difference in fluorescence intensities was observed between two groups. At the end of 1 h, nearly 1.5-fold higher cellular internalization was observed for PLGA-LHRH micelles than when compared with non-targeted PLGA micelles. The cellular uptake increased in a time-dependent manner, and nearly twofold increased uptake was seen with the targeted micelles. It could be expected that nanocarriers were internalized via endocytosis mechanism wherein the micelles will reside in the cytoplasmic region at first, following which, drug will be released and reach the nuclear or perinuclear region. It is well known that insufficient cellular internalization of a drug-loaded NPs leads to suboptimal intracellular drug concentration, less effective cancer therapy, and the potential for drug resistance. Similarly, higher cellular internalization will further improve the chemotherapeutic efficacy [23].

### Anticancer Effect of Non-Targeted and Targeted Micelles

**Fig. 5:**
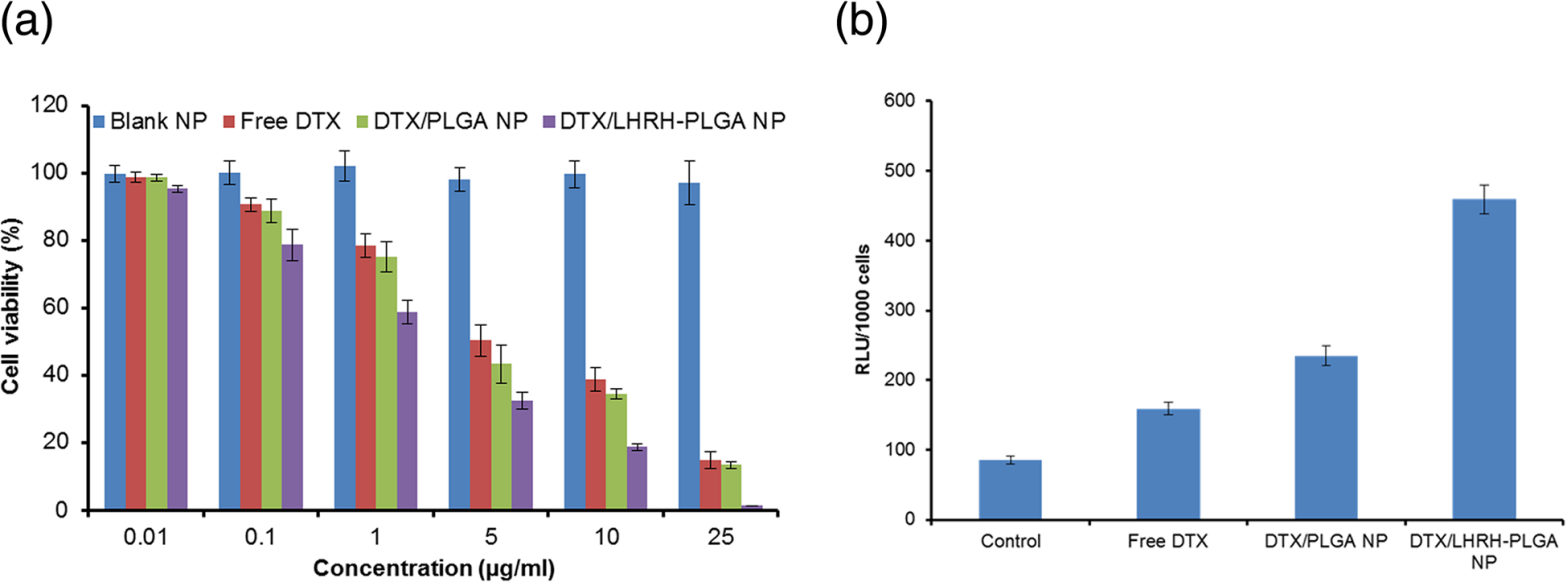
**a** Effect of free DTX, PLGA NP, and PLGA-LHRH micelles on the cellular viability of LNCaP prostate cancer cells. LNCap cells were treated with formulations and incubated for 24 h, and cell viability was determined by MTT assay. **b** Caspase-3 activity after treating with respective formulations

We next sought to determine the anticancer effect of free DTX, non-targeted DTX/PLGA NP, and DTX/PLGA-LHRH NP in LNCaP prostate cancer cells. At first, toxicity of blank polymer was tested at various concentrations. As seen (Fig. 5a), blank polymer showed showed high cell viability even at maximum tested concentrations indicating its non-toxic profile. Such delivery system will be very ideal of cancer-targeting purposes. Upon treatment with free DTX, DTX/PLGA, and DTX/PLGA-LHRH NP, a typical dose-dependent cytotoxicity was observed after 24 h incubation. Most notable observation is the anticancer effect of LHRH-conjugated micelles. It is clearly evident that LHRH conjugation to the NP surface greatly increased the cellular uptake and therefore increased the cell cytotoxicity. For example, at a dose of 5 μg/ml, the cell viability of DTX, DTX/PLGA, and DTX/PLGA-LHRH NP treated groups were 50.26, 43.28, and 30.34 % compared to that of control. Furthermore, IC50 value was calculated to quantify the individual effect of formulations on the cancer cells. It was observed that the IC50 value of DTX, DTX/PLGA, and DTX/PLGA-LHRH NP were 4.98, 3.95, and 1.28 μg/ml, respectively. The superior performance of targeted micelles was attributed to its specific affinity towards the LHRH receptors overexpressed in cancer cells and due to the enhanced cellular uptake. The low cytotoxicity exhibited by the non-targeted micelles may be attributed to their lower level of cellular uptake in comparison to that of the targeted micelles [24]. Our study confirms that the targeted delivery of bioactive agents results in enhanced efficacy in PCa therapy.

### Caspase-3 Activity

Consistent with the in vitro cytotoxicity assay, caspase-3 activity was evaluated in all aforementioned groups. As shown in Fig. 5b, LHRH-conjugated micelles induced significantly higher cell apoptosis than other groups. PLGA-LHRH exhibited threefold higher apoptosis than compared to free DTX treated cancer cells. Caspase-3 bring a crucial apoptosis marker; results suggest that surface conjugation of targeting ligand will be detrimental to cancer cells.

### Apoptosis Analysis

**Fig. 6:**
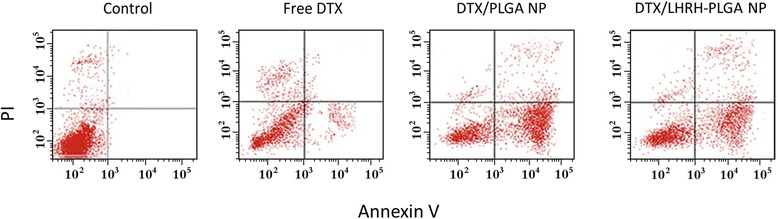
Annexin V/PI-based apoptosis analysis on prostate cancer cells

The apoptosis analysis was evaluated by means of Annexin-V/PI-based apoptosis kit. The apoptosis analysis was presented in Fig. 6 (*X* and *Y* axis shows the FITC and PI emissions). As seen, all the formulations exhibited considerable apoptosis of cancer cells after 24 h incubation. Free DTX induced nearly ~25 % of apoptosis (early and late apoptosis), whereas PLGA NP induced a ~56 % of apoptosis indicating the advantage of nanoparticles. Importantly, the presence of LHRH receptor greatly increased the fraction of apoptosis cells. As seen, approximately 62 % of cancer cells underwent apoptosis.

### Cell Cycle Analysis

**Fig. 7:**
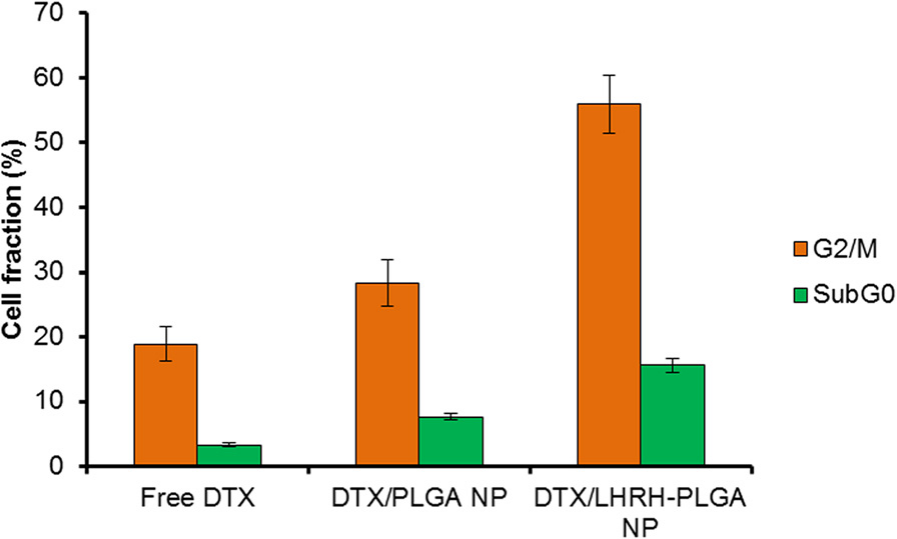
Cell cycle progression of LNCaP after treating with free DTX, PLGA NP, and PLGA-LHRH micelles

Flow cytometer analysis was carried out to determine the effect of targeted and non-targeted micelles on the cell cycle progression of LNCaP cells (Fig. 7). The results clearly showed that DTX and its formulations caused the cell death by a typical G2/M phase arrest. Specifically, LHRH-conjugated micelles induced threefold and twofold higher G2/M phase arrest than compared to free DTX or PLGA NP-treated groups. PLGA-LHRH exhibited nearly 60 % G2/M phase arrest than 30 % by PLGA NP. Notably, targeted micelles exhibited nearly ~15 % of cell death (subG0) than other groups. In general, DTX-loaded NP induce mitotic arrest by the release of drug which produces unstable microtubule, interfering with the mitotic spindle function, thereby arresting the cells in the G2/M phase of mitosis [25]. The results therefore suggest that targeted NPs kill the cancer cells more efficiently than the non-targeted NPs.

## Conclusions

In conclusion, we have successfully developed LHRH-conjugated PEGylated PLGA nanoparticles for the treatment of prostate cancers. The diblock polymers were synthesized and conjugated to LHRH by carbodiimide chemistry. A unique combination of targeted drug delivery and controlled drug release was proven to be effective against prostate cancer therapy. The PLGA-LHRH micelles possessed a uniform spherical shape with an average diameter of ~170 nm. The micelles exhibited a controlled drug release for up to 96 h which can minimize the non-specific systemic spread of toxic drugs during circulation while maximizing the efficiency of tumor-targeted drug delivery. The LHRH-conjugated micelles showed enhanced cellular uptake and exhibited significantly higher cytotoxicity against LNCaP cancer cells. We have showed that PLGA-LHRH induced greater caspase-3 activity indicating its superior apoptosis potential. Consistently, LHRH-conjugated micelles induced threefold and twofold higher G2/M phase arrest than compared to free DTX or PLGA NP-treated groups. Overall, results indicate that use of LHRH-conjugated nanocarriers could be an effective approach to target and kill prostate cancer cells. Additional studies, however, are required to further confirm the therapeutic potency of present delivery system.
